# Deacetylation of CHK2 by SIRT1 protects cells from oxidative stress-dependent DNA damage response

**DOI:** 10.1038/s12276-019-0232-4

**Published:** 2019-03-22

**Authors:** Jiyun Kwon, Suhee Lee, Yong-Nyun Kim, In Hye Lee

**Affiliations:** 1 Department of Life Science, Ewha Womans University, Seoul, South Korea; 2 Comparative Biomedicine Research Branch, Division of Translational Science, National Cancer Center, Goyang, Korea

**Keywords:** Cancer, Cancer metabolism

## Abstract

Growing evidence indicates that metabolic signaling pathways are interconnected to DNA damage response (DDR). However, factors that link metabolism to DDR remain incompletely understood. SIRT1, an NAD^+^-dependent deacetylase that regulates metabolism and aging, has been shown to protect cells from DDR. Here, we demonstrate that SIRT1 protects cells from oxidative stress-dependent DDR by binding and deacetylating checkpoint kinase 2 (CHK2). We first showed that essential proteins in DDR were hyperacetylated in Sirt1-deficient cells and that among them, the level of acetylated CHK2 was highly increased. We found that Sirt1 formed molecular complexes with CHK2, BRCA1/BRCA2-associated helicase 1 (BACH1), tumor suppressor p53-binding protein 1 (53BP1), and H2AX, all of which are key factors in response to DNA damage. We then demonstrated that CHK2 was normally inhibited by SIRT1 via deacetylation but dissociated with SIRT1 under oxidative stress conditions. This led to acetylation and activation of CHK2, which increased cell death under oxidative stress conditions. Our data also indicated that SIRT1 deacetylated the K235 and K249 residues of CHK2, whose acetylation increased cell death in response to oxidative stress. Thus, SIRT1, a metabolic sensor, protects cells from oxidative stress-dependent DDR by the deacetylation of CHK2. Our findings suggest a crucial function of SIRT1 in inhibiting CHK2 as a potential therapeutic target for cancer treatment.

## Cancer therapy: Limiting responses to DNA damage

Protein interactions that protect cells from excessive responses to DNA damage may help improve cancer treatments. Normal metabolic processes and environmental factors such as exposure to radiation can damage DNA within cells. Excessive responses to severe damage can result in uncontrolled cell division and tumor growth. The metabolic protein SIRT1 is known to help protect cells during responses to DNA damage. In Hye Lee at Ewha Womans University in Seoul, South Korea, and co-workers examined the interactions between SIRT1 and a stimulator of responses to DNA damage, checkpoint kinase 2 (CHK2). Under normal conditions, SIRT1 physically binds with CHK2 and limits its activity. Under oxidative stress, for example during certain cancer treatments, CHK2 dissociates from SIRT1, and its enzymatic activity is increased. Therefore, treatments that increase SIRT1 levels could deactivate CHK2 and protect cells.

## Introduction

Metabolism and the DNA damage response (DDR) mechanism are essential biological processes for the survival of animals and cells but are generally considered to be two distinct processes. However, a number of recent studies have suggested extensive crosstalk between DDR and metabolism. Ataxia telangiectasia mutated (ATM) and p53, essential factors for DDR, are known crucial regulators of normal metabolism. For instance, insulin secretion is impaired in *Atm* knockout mice, and *ApoE* knockout further perturbs metabolism in *Atm* knockout mice, resulting in impaired glucose metabolism and atherosclerosis^1,2^. *p53* loss of function mutations can cause metabolic dysfunction, including glucose intolerance and insulin resistance^3–5^. Conversely, the dysfunction of molecular components in metabolism exerts effects on DDR. Deficiency in Atg7, an essential autophagy component, elevates DDR through the generation of mitochondrial reactive oxygen species (ROS)^6^. Additionally, DDR is potentiated by Atg5 deficiency^7^. Nevertheless, the molecular connection between metabolism and DDR remains incompletely understood.

Sirtuins are protein deacetylases that affect important physiology and pathology mechanisms, including aging, cancer, neurodegeneration, and metabolism^8–11^. Recent studies have indicated that sirtuins regulate DDR and redox signaling^12^. Sirtuins protect cells from ROS-induced damage and regulate the expression of key factors, including nuclear factor E2-related factor 2 (NRF2), in response to oxidative stress^13,14^. When cells are under stress conditions, ROS production is increased, and the sirtuin co-factor NAD^+^ activates various sirtuins. Additionally, sirtuins regulate the activity of antioxidant response element (ARE), which regulates the transcription of pro- and antioxidant genes. This contributes to the maintenance of redox signaling cascades and redox homeostasis by balancing antioxidant enzymes and pro-oxidant radicals^12^. Furthermore, the deletion of sirtuins elicits increases in DDR. However, the molecular mechanisms by which sirtuins regulate DDR remain largely unexplored.

CHK2 is a key regulator of DDR. CHK2 is the target of the DDR sensor kinase ATM in response to genotoxic stress, such as ROS, ultraviolet radiation, and chemotherapeutic reagents. It is generally believed that CHK2 is activated by the ATM kinase^15,16^. Upon sensing any of a number of stresses, ATM phosphorylates and activates the transducer kinase CHK2, which in turn phosphorylates p53, a CHK2 target. Activated p53 can result in cell fate decision, including cell death or G2/M arrest. CHK2 also regulates cell cycle control and maintains genome stability^17^.

Here, we show a new mechanism by which SIRT1 regulates the oxidative stress-dependent DDR. In particular, we found that SIRT1 physically interacted with multiple essential proteins involved in responses to DNA damage, including CHK2, BACH1, 53BP1, and H2AX. Among these proteins, we showed that CHK2 was a direct deacetylation target of SIRT1. We found that SIRT1 deficiency increased the acetylation and activity of CHK2 under oxidative stress conditions. SIRT1 HY, an inactive mutant form, also stimulated CHK2 activity under oxidative stress conditions, but wild-type SIRT1 did not. Additionally, SIRT1 HY rescue in SIRT1 knockout cells failed to recover cell survival in response to oxidative stress. Moreover, the CHK2 deacetylation mimic K235R/K249R protein was constitutively inactive and increased cell survival in response to oxidative stress. Taken together, our data suggest that SIRT1 inhibits CHK2 by deacetylation to protect cells from DDR.

## Materials and methods

### Cell culture

HeLa and HCT116 cells were cultured at 37 °C in DMEM and McCoy’s 5 A media (WELGENE, South Korea), respectively, including 10% fetal bovine serum (FBS, Young In Frontier, South Korea) and antibiotic-antimycotic solution (100 U/ml penicillin, 100 μg/ml streptomycin and 250 ng/ml amphoteric B) (WELGENE). Primary mouse embryonic fibroblasts (MEFs) were isolated from *SIRT1* knockout mice^18^ using a standard method and cultured in DMEM containing 10% fetal bovine serum and the antibiotic-antimycotic solution. Differences in cell death levels were confirmed by using three or more independent MEF cell isolates.

### DNA construction

For mammalian cell expression, CHK2 WT (amino acids 1-543) was generated by PCR amplification of pENTR-CHK2 using primers, 5′-GCG AAT TCG ATG TCT CGG GAG TCG-3′ and 5′-GGC TGG TAC CGT TCA CAA CAC AGC-3′. Amplified fragments were ligated into the EcoRI/KpnI sites of the 3XFlag-CMV-7.1 plasmid (Sigma-Aldrich, St. Louis, MO, USA) and subsequently verified by sequencing. CHK2 mutants that included K235R/K249R CHK2 and K235Q/K249Q CHK2 were generated by using a QuickChange Site-directed mutagenesis kit (Agilent Technologies, Santa Clara, CA, USA) following the manufacturer’s protocols. CHK2 mutant DNA was amplified using the following primers sets: for K235R/K249R CHK2, 5′-AAA GTA GCC ATA AGG ATC ATC AGC AAA AGG AAG-3′, 5′-CTT CCT TTT GCT GAT GAT CCT TAT GGC TAC TTT-3′, 5′-GAA AGC CAG CCT TAC CTC TCC ACA GGC ACC-3′, and 5′-GGT GCC TGT GGA GAG GTA AGG CTG GCT TTC-3′; and for K235Q/K249Q, 5′-AAA GTA GCC ATA CAG ATC ATC AGC AAA AGG AAG-3′, 5′-CTT CCT TTT GCT GAT GAT CTG TAT GGC TAC TTT-3′, 5′-GAA AGC CAG CTG TAC CTC TCC ACA GGC ACC AC, and 5′-GTG GTG CCT GTG GAG AGG TAC AGC TGG CTT TC-3′. All constructs were confirmed by sequencing.

### Cell viability

As described in a previous report^6^, 6–8 × 10^4^ cells of either WT or *SIRT1* knockout MEFs/HeLa cells/HCT116 cells were placed in 12-well plates. Where indicated, 48 h after seeding, cells were transferred from complete media to Hank’s Balanced Salt Solution (HBSS, WELGENE, South Korea) with 1 or 3 mM of H_2_O_2_ for 1 h. Cell viability was determined by trypan blue exclusion. Similar experiments were performed using *SIRT1*-deficient HeLa cells. HeLa cell viability was determined by XTT assay (Sigma-Aldrich, St. Louis, MO, USA) based on the manufacturer’s protocols. Data are reported as single experimental results from triplicates representative of more than three independent experiments.

### Transfection

As described previously^6,18^, HCT116 or HeLa cells were routinely transfected with Effectene (Qiagen, Hilden, Germany) according to the manufacturer’s recommendations. To assess cell viability, 12-well plates of HCT116 cells were transfected with 0.3 μg of vector or CHK2 variants. Immortalized *SIRT1* KO MEFs were transfected with 1 µg of Flag-tagged SIRT1 WT or HY mutant constructs and analyzed 24 h after transfection. To measure the acetylation of CHK2 constructs, 100-mm dishes of HCT116 cells were transfected with 3 µg of the 3XFlag-tagged CHK2 constructs using Effectene and were assayed 24 h after transfection. For transient *SIRT1* siRNA knockdown experiments, HeLa cells or HCT116 cells were transfected using either 300 nM of a siRNA targeting *SIRT1* (Dharmacon, Lafayette, CO, USA) or a corresponding control nontargeting siRNA (scrambled). Cells were analyzed 48 h after transfection.

### Immunoprecipitation and immunoblotting

As described in a previous report^18^, the lysates (1–2 mg) of HeLa cells, HCT116 cells or MEFs were incubated with indicated antibodies (1–2 μg) at 37 °C overnight; 40 μl of protein G-sepharose (GE healthcare, Chicago, IL, USA) was then added. After 2 h, immune complexes were washed three times with lysis buffer [50 mM Tris pH 7.4, 1% Triton X-100, 0.5% NP-40, 150 mM NaCl, protease inhibitor cocktail (Sigma-Aldrich, St. Louis, MO, USA), PhosphoSTOP (Sigma-Aldrich, St. Louis, MO, USA) and 10% glycerol]. After sampling, the immune complexes were separated by SDS-PAGE and transferred to a nitrocellulose membrane. Membranes were immunoblotted with specific primary antibodies against BACH1 (Sigma-Aldrich, St. Louis, MO, USA), 53BP1 and H2AX (Novus Biologicals, Centennial, CO, USA), SIRT1 (Millipore, Burlington, MA, USA or Santa Cruz Biotechnology, Dallas, TX, USA), or CHK2 (BD Biosciences, San Jose, CA, USA) followed by appropriate horseradish peroxidase-conjugated secondary antibodies (Young In Frontier, South Korea). Bands were visualized by enhanced chemiluminescence (Young In Frontier, South Korea).

### Assessment of DDR

For γ-H2AX staining (phosphorylation of histone protein H2AX on serine 139) or 53BP1 staining, 7 × 10^4^ HeLa cells grown on Lab-tek II (Thermo Fisher Scientific, Waltham, MA, USA) dishes were genetically knocked down, as previously described^6,18^. Forty-eight hours later, cells were washed with cold PBS, fixed with 4% paraformaldehyde in PBS for 10 min at room temperature, and permeabilized with 0.5% Triton X-100 in PBS. Nonspecific sites were blocked by treating the cells with PBS containing 1% bovine serum albumin and 0.05% Triton X-100 for 1 h. The cells were incubated with primary antibodies against γ-H2AX (Novus Biologicals, 1:300 in 1% BSA) or 53BP1 (Novus Biologicals, 1:300 in 1% BSA) overnight at 4 °C and washed with PBS. Cells were incubated with AlexaFluor 594 goat anti-rabbit IgG (1:1000, Thermo Fisher Scientific) for 1 h, and washed with PBS. Cells were counterstained with 300 nM DAPI (Thermo Fisher Scientific) for 5 min and subsequently washed twice with PBS. Images were captured using a confocal laser scanning microscope (Carl Zeiss, Oberkochen, Germany). Three independent experiments were performed in duplicate per condition; a minimum of 50 random cells were counted per condition. Data are reported as the mean ± SD.

## Results

### SIRT1 regulates the acetylation status of important proteins of DDR including CHK2

**Fig. 1:**
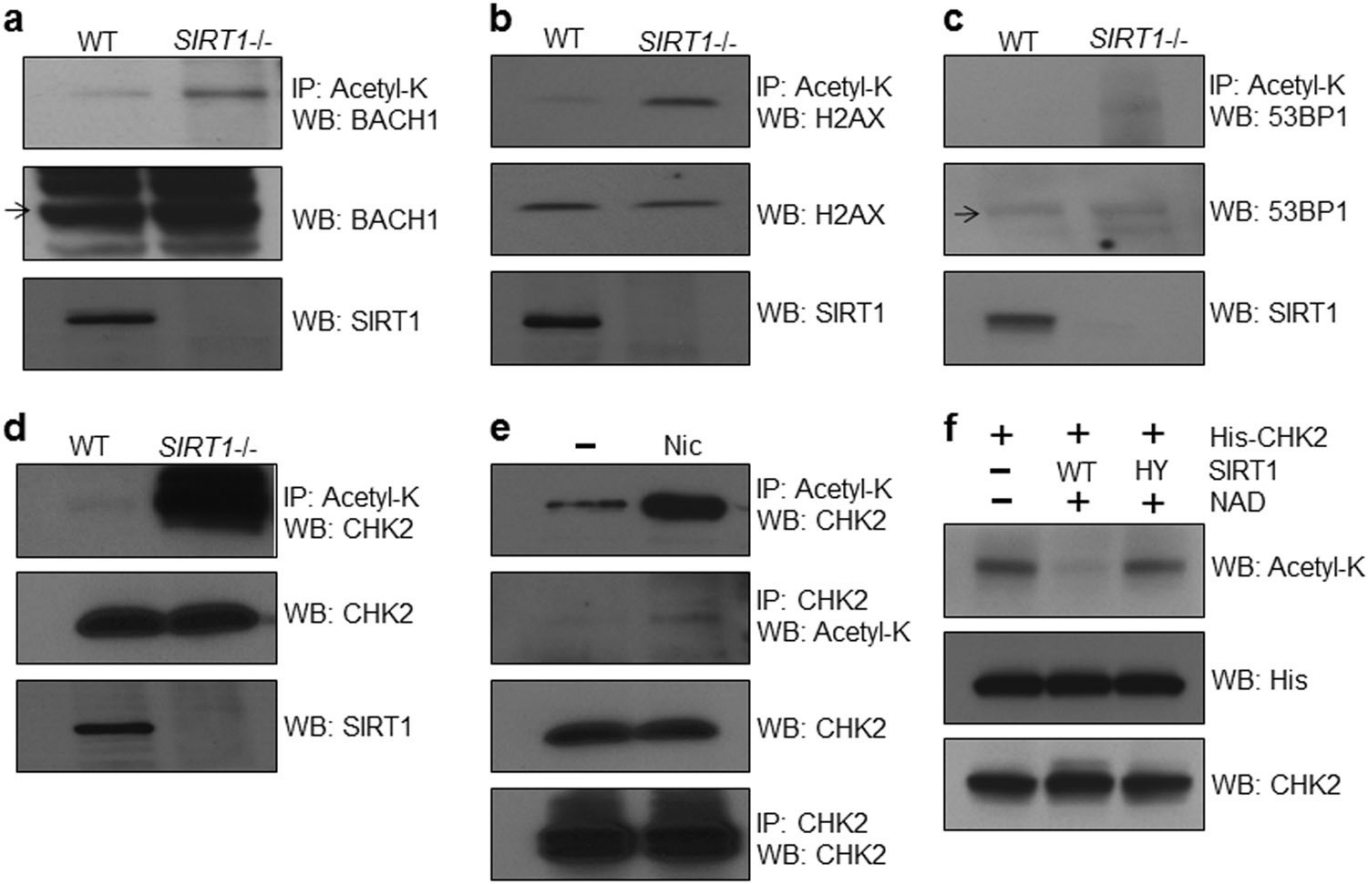
SIRT1 regulates the acetylation status of the molecular complexes in response to DNA damage. **a**–**c** The acetylation levels of BRCA1/BRCA2-associated helicase 1 (BACH1), H2AX, and tumor suppressor p53-binding protein 1 (53BP1) were increased in *SIRT1* knockout (KO) mouse embryonic fibroblasts (MEFs). **d** The acetylation level of CHK2 (Checkpoint Kinase 2) was highly elevated in the *SIRT1*-deficient cells. **e** Treatment with nicotinamide (Nic) increased the acetylation level of CHK2 in HeLa cells. **f** Purified CHK2 in HeLa cells was deacetylated by purified SIRT1 WT but not by SIRT1 HY in an NAD^+^-dependent manner. IP immunoprecipitation, WB Western blot, NAD nicotinamide adenine dinucleotide. All western blots in figures are representative of three independent experiments

Previous reports have shown that SIRT1 deacetylates p53, a key factor of apoptosis after DNA damage, and that CHK2 interacts with SIRT1^19,20^. We therefore sought to determine whether SIRT1 targeted and deacetylated important proteins that mediated DDR. We found that the acetylation levels of BACH1, H2AX, and 53BP1 all modestly increased in *SIRT1* knockout mouse cells (Fig. 1a–c). In contrast, we observed that the acetylation level of CHK2 dramatically increased in *SIRT1*-deficient mouse cells (Fig. 1d). We therefore focused our subsequent analysis on the connection between SIRT1 and CHK2. Consistent with a role for sirtuins in CHK2 acetylation, treatment of HeLa cells with nicotinamide (Nic), a pharmacological inhibitor of sirtuins, increased the level of CHK2 acetylation (Fig. 1e). We tested whether SIRT1 directly deacetylated CHK2 by means of an in vitro deacetylation assay.

We purified SIRT1 wild-type (WT) and a deacetylase-inactive mutant form (HY) of SIRT1 as well as His-tagged CHK2. Purified SIRT1 WT, but not the catalytically inactive HY form, deacetylated CHK2 in an NAD^+^-dependent manner (Fig. 1f). Together, these results suggested that SIRT1 directly deacetylated CHK2.

### SIRT1 dissociates with CHK2 in response to oxidative stress

**Fig. 2:**
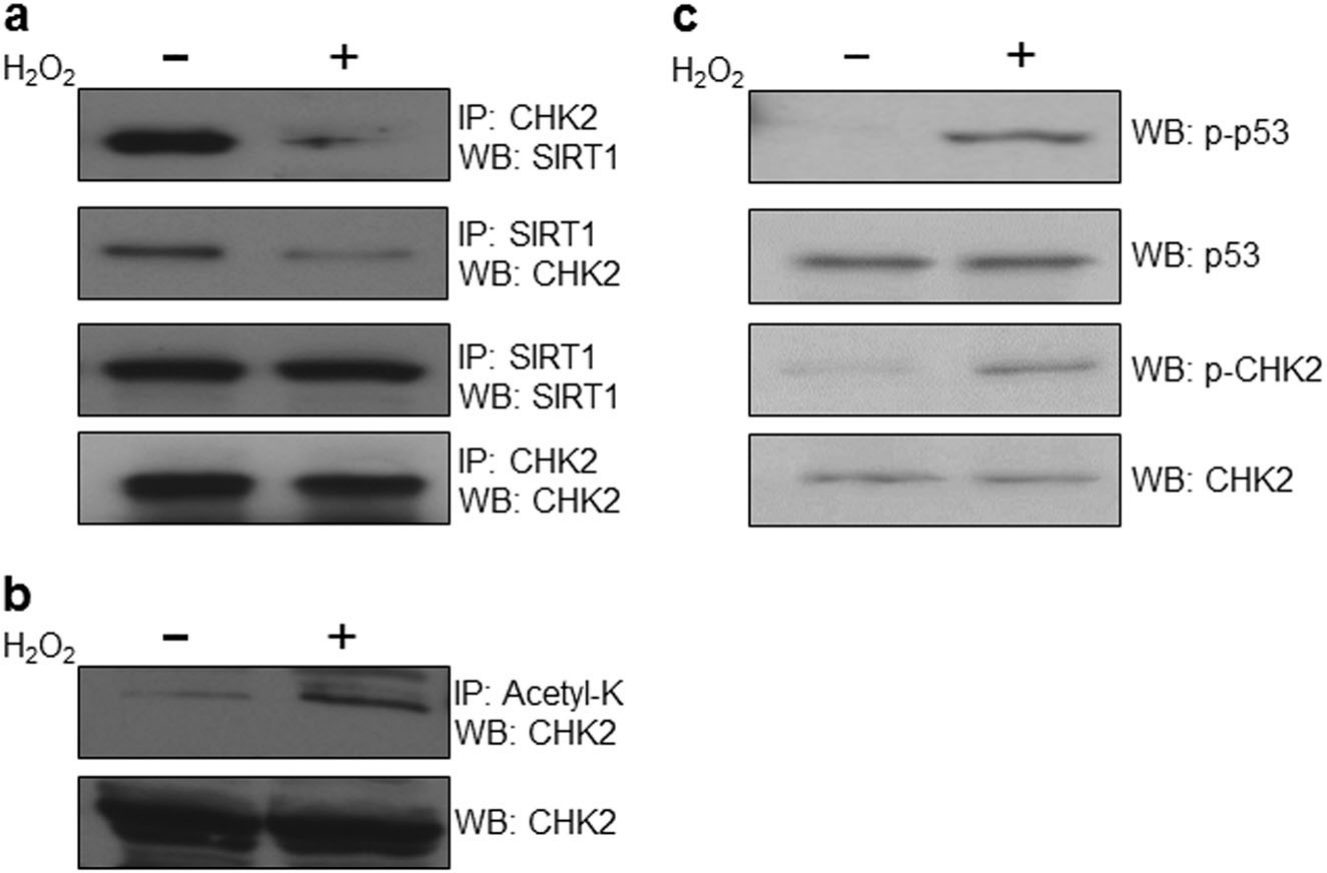
SIRT1 dissociates with CHK2 for its activation upon oxidative stress. **a** SIRT1 bound CHK2 under control conditions but dissociated with CHK2 after treatment with 1 mM of H_2_O_2_ in HeLa cells. **b** The acetylated CHK2 level was elevated in response to oxidative stress in HeLa cells. **c** DDR pathways were activated upon oxidative stress. The phosphorylation levels of CHK2 (p-CHK2) and p53 (p-p53) were highly increased in response to oxidative stress in HCT116 cells. IP immunoprecipitation, WB Western blot. Western blots in figures are representative of more than three independent experiments

Next, we sought to further examine the interaction between SIRT1 and CHK2. We found that SIRT1 dissociated from CHK2 in response to hydrogen peroxide (H_2_O_2_) (Fig. 2a). Therefore, the interaction between SIRT1 and CHK2 appears to be regulated by oxidative stress. We then asked whether oxidative stress altered the acetylation status of CHK2 and found that the level of acetylated CHK2 increased under oxidative stress conditions (Fig. 2b). Taken together, these data suggest that SIRT1 deacetylates CHK2 under basal conditions, but oxidative stress results in a concomitant dissociation of the CHK2-SIRT1 complex and a corresponding increase in CHK2 acetylation. Consistent with previous reports^21,22^, we found that oxidative stress potentiated DDR, as evidenced by increased levels of phospho-p53 and phospho-CHK2 (Fig. 2c).

### *SIRT1* deficiency increases DDR-mediated cell death and CHK2 activity under oxidative stress conditions

**Fig. 3:**
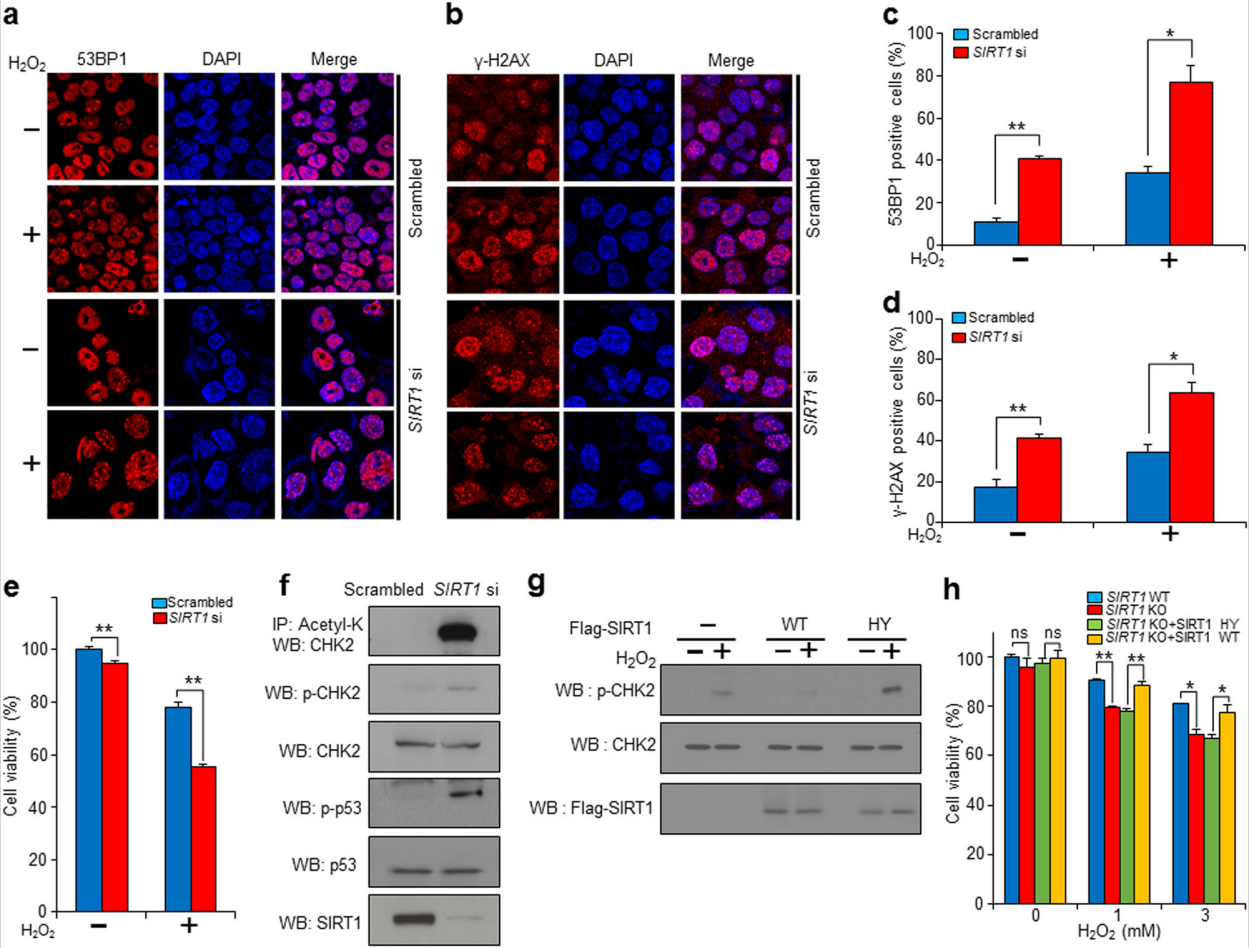
Genetic inhibition of *SIRT1* increases DDR-mediated cell death in oxidative stress conditions via upregulation of CHK2 activity. **a**, **b** Representative images of the levels of DNA damage in *SIRT1*-deficient HeLa cells with/without oxidative stress. They were assessed by 53BP1 or phospho-H2AX staining. **c**, **d** *SIRT1* deficiency potentiated DDR. Both 53BP1 and phospho-H2AX foci formation was increased in *SIRT1*-deficient HeLa cells. Images were captured using a confocal laser scanning microscope from three independent experiments performed with duplicates per condition, counting approximately a minimum of 50 cells per condition (mean ± SD, *P < 0.05, **P < 0.01, Student’s *t*-test). **e** The viability of *SIRT1*-deficient HeLa cells was further decreased compared with control (scrambled) cells, after treatment with H_2_O_2_ for 1 h. **f** The levels of acetylated CHK2, phospho-CHK2 (p-CHK2) and phospho-p53 (p-p53) were increased in *SIRT1*-knockdown HCT116 cells. **g** Inactive SIRT1 mutants (SIRT1 HY) potentiated CHK2 activity by increasing p-CHK2 levels in HCT116 cells. **h** *SIRT1* deficiency (KO) increased cell death under oxidative stress conditions. The oxidative stress-mediated cell death in mouse embryonic fibroblasts was rescued by overexpression of SIRT1 WT but not by overexpression of SIRT1 HY. IP immunoprecipitation, WB western blot. Data represent the mean ± SD (*P < 0.05, **P < 0.01, ns: not significant, Student’s *t*-test). Shown are single experimental results from triplicates that are representatives of more than three independent experiments

Because reactive oxygen species (ROS) are well known DDR inducers^21^, we sought to determine whether SIRT1 deficiency increased DDR under oxidative stress conditions. To assess this question in a more physiologically relevant system, we used siRNA to knockdown SIRT1 in HeLa cells. We then examined the markers of DDR, in particular, the foci of 53BP1 or γ-H2AX, in the presence or absence of oxidative stress. We found that the siRNA knockdown of *SIRT1* significantly elevated the number of 53BP1 foci and γ-H2AX foci in HeLa cells (Fig. 3a–d).

Next, we investigated whether SIRT1 influenced CHK2 activity under oxidative stress conditions. We measured the levels of phospho-CHK2 and phospho-p53 to determine CHK2 activity upon treating control and *SIRT1*-deficient HCT116 cells with H_2_O_2_. The phosphorylation levels of p53 and CHK2 increased after treatment with H_2_O_2_, and the increases were more pronounced when *SIRT1* was knocked down (Fig. 3f). Consistent with data using *SIRT1*-deficient mouse cells (Fig. 1d), we found that the acetylation of CHK2 highly increased in *SIRT1*-knockdown human cells (Fig. 3f). We also tested whether the deacetylase activity of SIRT1 was necessary for the activation of CHK2 under oxidative stress conditions. By using SIRT1 WT and SIRT1 inactive mutant (SIRT1 HY) (Fig. 3g), we found that overexpression of SIRT1 WT decreased the phosphorylation of CHK2 compared with vector control in response to oxidative stress. However, the phospho-CHK2 level highly increased in HCT116 cells in which SIRT1 HY mutants were overexpressed (Fig. 3g). Our results suggested that CHK2 activity was potentiated by *SIRT1* deficiency and that the deacetylase activity of SIRT1 downregulated CHK2 under oxidative stress conditions.

We then determined the physiological role of SIRT1-mediated deacetylation of CHK2 in DDR by treating *SIRT1*-deficient human cells and mouse embryonic fibroblasts (MEFs) with H_2_O_2_. We found that *SIRT1*-deficient HeLa cells were more susceptible to cell death in response to excessive ROS than control cells (Fig. 3e). The viability of control cells decreased up to 20% upon treatment with H_2_O_2_. In contrast, *SIRT1*-knockdown cells displayed 40% cell death (Fig. 3e). Thus, SIRT1 depletion appeared to increase sensitivity to oxidative stress. We also observed that *SIRT1* knockout (KO) mouse embryonic fibroblasts (MEFs) compared with *SIRT1* WT MEFs were more susceptible to cell death when we treated MEF cells with H_2_O_2_. We tested whether the deacetylase activity of SIRT1 was required for cell survival under oxidative stress conditions. We found that the overexpression of SIRT1 WT restored cell survival in *SIRT1* knockout MEFs but a deacetylase-inactive mutant SIRT1 (SIRT1 HY) did not (Fig. 3h). Thus, the deacetylase activity of SIRT1 appeared to mediate cell survival under oxidative stress.

### K235 and K249 of CHK2 are potential deacetylation sites of SIRT1

**Fig. 4:**
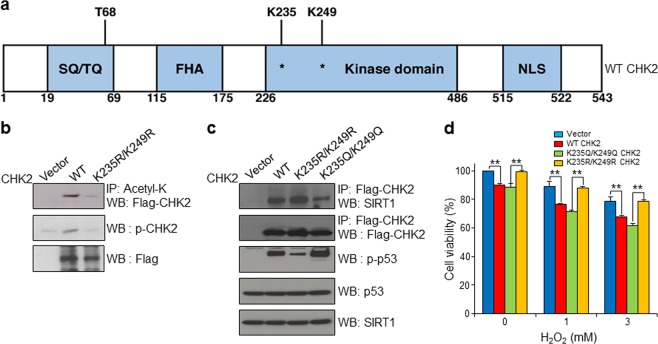
K235 and K249 residues of CHK2 are deacetylated by SIRT1. **a** Domain structure of CHK2. **b** The acetylation and phosphorylation levels of CHK2 were largely decreased by K235R/K249R substitution (KR) of CHK2 in HCT116 cells. **c** K235Q/K249Q changes in CHK2 decreased the interaction between CHK2 and SIRT1 and increased the level of p-p53 compared with WT CHK2 in HCT116 cells. **d** Under oxidative stress conditions, WT CHK2 and K235Q/K249Q CHK2 enhanced cell death but K235R/K249R CHK2 did not in HCT116 cells. IP immunoprecipitation, WB western blot. Data represent the mean ± SD (*P < 0.05, **P < 0.01, ns: not significant, Student’s *t*-test). Shown are single experimental results from triplicates that are representatives of three independent experiments

We sought to identify amino acid residues in CHK2 that are deacetylated by SIRT1. We found that K235 and K249 were potential acetylation sites of CHK2 using PhosphositePlus and prediction of acetylation on internal lysines (PAIL) (https://www.phosphosite.org/, http://bdmpail.biocuckoo.org/) (Fig. 4a). We then generated an acetylation mimic mutant K235Q/K249Q CHK2 construct and a deacetylation mimic mutant K235R/K249R CHK2 construct. We found that K235R/K249R substitution robustly decreased the acetylation level of CHK2 (Fig. 4b). We also showed that the active phospho-CHK2 level was decreased by K235R/K249R substitution compared with WT (Fig. 4b).

We further analyzed the biological activity of mammalian CHK2 variants. Increased CHK2 expression stimulated CHK2 activation (Fig. 4b) and increased the phosphorylation of p53, the target of CHK2. Overexpression of K235Q/K249Q CHK2 further elicited the phosphorylation of p53 but K235R/K249R CHK2, a potential inactive protein, did not (Fig. 4c). However, both WT CHK2 and K235R/K249R CHK2 bound to SIRT1 (Fig. 4c). This interaction was substantially decreased by K235Q/K249Q changes in CHK2. Thus, SIRT1 appeared to bind to CHK2 to regulate the phosphorylation of CHK2 and p53. These data suggested that changes to the acetylation statuses at K235 and K249 regulated the activity of CHK2. We then determined whether the acetylation status of CHK2 influenced cell viability under oxidative stress conditions by using K235Q/K249Q CHK2 and K235R/K249R CHK2 mutants. We found that the overexpression of WT CHK2 in HCT116 cells increased cell death in response to H_2_O_2_ (Fig. 4d). We showed that the acetylation mimic K235Q/K249Q CHK2 further increased cell death under oxidative stress conditions (Fig. 4d). In contrast, the deacetylation mimic K235R/K249R CHK2 recovered cell death to the level observed in control cells (Fig. 4d). Taken together, these data suggested that the inactivation of CHK2 by SIRT1-mediated deacetylation at K235 and K249 was essential for preventing oxidative stress-dependent cell death.

## Discussion

Metabolic signaling pathways and DDR are closely associated, but factors that connect these two processes remain poorly understood. In this report, we showed that SIRT1 protected cells from oxidative stress-dependent DDR by binding to and deacetylating CHK2. We found that oxidative stress changed molecular complex formation and dissociation of SIRT1 with CHK2, which plays essential roles in DDR. Under normal conditions, SIRT1 acts as a scaffold by forming complexes with important proteins in DNA damage signaling pathways, including BACH1, p53BP1, and H2AX (Sup Fig. 1) as well as CHK2. We proposed that these signaling proteins efficiently transferred DNA damage signals to downstream proteins in stressed cells. Compared with dissociations between SIRT1 and CHK2 under oxidative stress conditions (Fig. 2a), interactions among BACH1, 53BP1, and SIRT1 did not change upon treatment with H_2_O_2_ (Sup Fig. 1a and 1b). We showed that CHK2 specifically dissociated from SIRT1 under oxidative stress conditions. We also demonstrated that H2AX bound SIRT1 more strongly under oxidative stress (Sup Fig. 1c). Thus, SIRT1 seemed to regulate the function of H2AX differently from the regulation of CHK2. It would be interesting to identify new signaling pathways that connect H2AX to SIRT1 by modulating H2AX by SIRT1.

We also showed that CHK2 was the substrate of SIRT1 deacetylase and that acetylated CHK2 under oxidative stress conditions increased CHK2 own activity. This led to deceased cell survival under oxidative stress conditions. Our data also demonstrated that SIRT1 deacetylated and inhibited CHK2 activity, which resulted in the prevention of cells from oxidative stress-dependent cell death. We observed that SIRT1 HY, an inactive mutant form, stimulated the activity of CHK2 by increasing phosphorylation (Fig. 3g). Additionally, SIRT1 deacetylase activity was required for recovering the survival of SIRT1 knockout (KO) mouse embryonic fibroblast cells under oxidative stress conditions (Fig. 3h). Furthermore, the activity of SIRT1 was necessary for the deacetylation of CHK2 to inhibit its activity (Fig. 1f). These results suggested that the deacetylase activity of SIRT1 was essential for CHK2 regulation.

We believe that our findings will be useful for developing strategies for treating cancer by using ionizing radiation (IR) and/or chemotherapeutic drugs. Here, we showed that 5-fluorouracil (5-FU), an anti-cancer drug, dissociated SIRT1-CHK2 complexes with increased CHK2 activity (Sup Fig. 2). We also found that the dissociation between SIRT1 and CHK2 occurred in response to 5-FU (Sup Fig. 2). Additionally, treatment with 5-FU increased the level of the active phosphorylated form of CHK2 (Sup Fig. 2). These data suggested that SIRT1 dissociated from CHK2 as a general response to DNA damage and indicated that the dissociation between SIRT1 and CHK2 could serve as a potential marker for DDR.

**Fig. 5:**
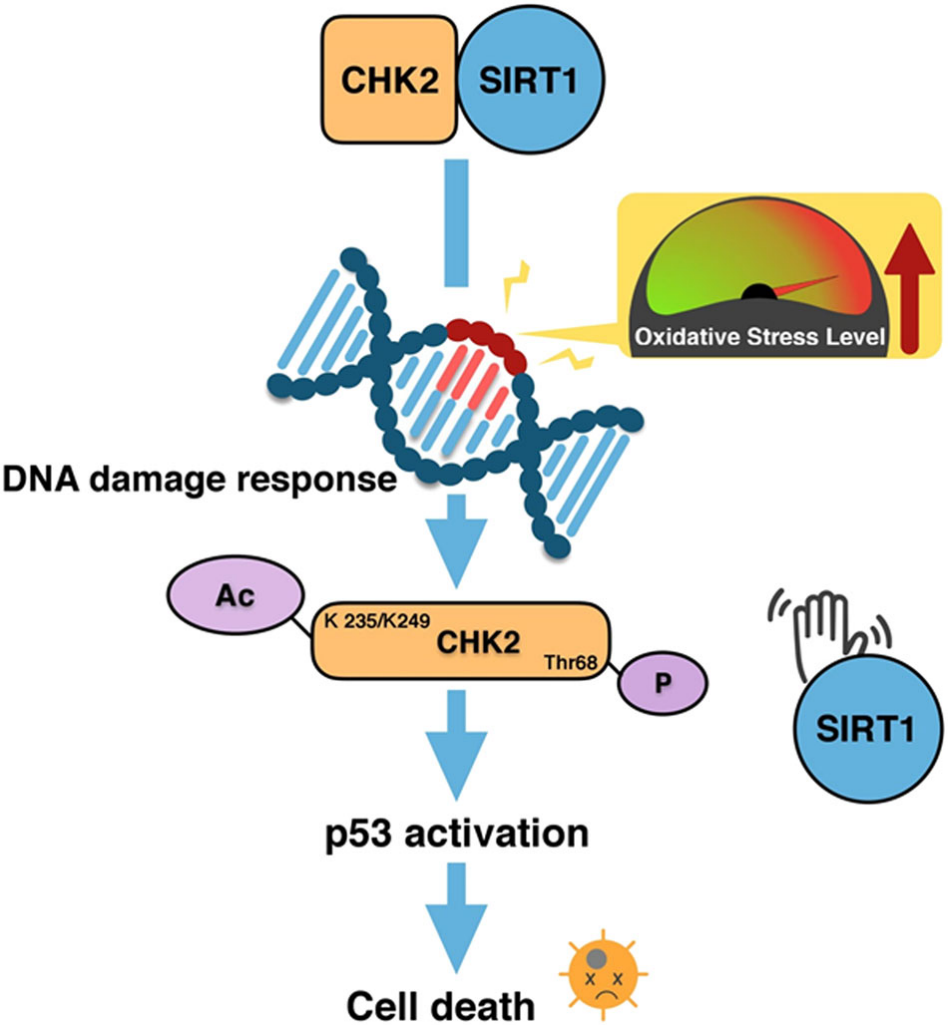
Suggested model. Under normal conditions, SIRT1 binds to CHK2 and inhibits its activity through deacetylation. Oxidative stress initiates DDR pathways, which leads to dissociation of SIRT1 from CHK2, and this in turn increases the acetylation and the activity of CHK2. The phosphorylation and acetylation of CHK2 potentiates p53 activity, which results in cell death upon oxidative stress

Our study also provided new clues for a combination of cancer therapies using SIRT1 regulators and CHK2 inhibitors. Previous studies reported that SIRT1 has dual effects on tumors^23–28^. SIRT1 has been shown to be a tumor suppressor or a tumor promoter based on the status of p53 and depending on the types and stages of cancers^23,24^. In our current work, we discovered intracellular mechanisms by which SIRT1 negatively regulated CHK2 in human and mouse cells (Fig. 5). Indeed, several previous studies reported the use of CHK2 inhibitors in clinical trials for the treatment of cancers^29–36^. Many previous reports have shown that SIRT1 is a tumor suppressor. SIRT1-activating compounds (STACs) have been developed and used in clinical trials^37–43^. We believe that our findings are novel because they suggest the possibility that increased SIRT1 activity, together with treatment with an inhibitor of CHK2 that can reduce tumor pathology.

A recent report showed that the depletion of CHK2 inhibited associations between SIRT1 and DBC1, which reduced SIRT1 activity by decreasing the acetylation of p53 and p53-dependent apoptosis. CHK2 forms a basal complex with DBC1 and with SIRT1, but these proteins are not substrates of CHK2. Phosphorylation of REGγ by CHK2 increases the interaction between REGγ and inhibits DBC1 and SIRT1 in DDR, leading to increases in the acetylation of p53 and cell death^20^.

Here, we found that SIRT1 targeted and regulated the activity of CHK2 by deacetylation in an oxidative stress-dependent manner. The enzymatic activity of CHK2 is known to be regulated by ATM-mediated phosphorylation^15,16^. Our findings indicated a new mechanism that regulates the activity of CHK2 by changing its acetylation status by SIRT1 (Fig. 5). We showed that the dissociation of SIRT1 from CHK2 further increased the serine/threonine kinase activity of CHK2. This suggested that CHK2 was activated by acetylation and phosphorylation. It would be interesting to determine the acetylation and phosphorylation levels of CHK2 in cancer patients treated with therapeutic drugs or irradiation. Moreover, it would be useful to identify K235/K249 changes of CHK2 in cancer patients as a diagnosis tool for cancer.

## Supplementary information

Supplementary Figure Legends

Supplementary Figure 1

Supplementary Figure 2
