# Supplementary material for: Deacetylation of CHK2 by SIRT1 protects cells from oxidative stress-dependent DNA damage response
Source: Exp Mol Med. 2019 Mar 22;51(3):1–9. doi: 10.1038/s12276-019-0232-4 (PMC6430805; doi:10.1038/s12276-019-0232-4)
Supplement: Supplementary file 1 — Supplementary Figure Legends [file 12276_2019_232_MOESM1_ESM.docx]

**Deacetylation of CHK2 by SIRT1 protects cells from oxidative stress-dependent DNA damage response**

**Jiyun Kwon^1^, Suhee Lee^1^, Yong-Nyun Kim^2^ and In Hye Lee^1^***

^1^Department of Life Science, Ewha Womans Univeristy, Seoul, South Korea, and ^2^Comparative Biomedicine Research Branch, Division of Translational Science, National Cancer Center, Korea

*Corresponding author: In Hye Lee

Department of Life Science, Ewha Womans University, 52, Ewhayeodae-gil, Seodaemun-gu, Seoul, South Korea 03760

E-mail: lih3026@ewha.ac.kr

Phone: 82-2-3277-3032

Fax: 82-2-3277-3760

This work was supported by grants from the National Research Foundation of Korea (NRF-2016R1D1A1B04934603, NRF-2014R1A1A3051320, and NRF-2014M3A9D8034459)

**Supplementary Information**

**Figure legends**

**Supplementary Fig1. SIRT1 makes molecular machinery with BACH1, 53BP1 and H2AX under normal condition.** **a-b** SIRT1 interacted with BACH1 or 53BP1 with or without H_2_O_2_ treatment in HeLa cells. **c** Stimulation of H_2_O_2_ in HeLa cells increased interaction between SIRT1 and H2AX. IP: immunoprecipitation. WB: Western blot. Western blots in figures are representative of more than three independent experiments.

**Supplementary Fig2. SIRT1 dissociates with CHK2 in response to 5-fluorouracil (5-FU).** Under normal condition, SIRT1 made a stable complex with CHK2. However, CHK2 strongly dissociated with SIRT1 with treatment of DMSO (0μg/ml of 5-FU) or 2μg/ml of 5-FU (Sigma) for 24hr in HCT116 cells. Treatment of HCT116 cells with anti-cancer drug 5-FU increased DNA damage and enhanced CHK2 activity. IP: immunoprecipitation. WB: Western blot. Western blots in figures are representative of three independent experiments.
