# Supplementary figures and images for: Deacetylation of CHK2 by SIRT1 protects cells from oxidative stress-dependent DNA damage response
Source: Exp Mol Med. 2019 Mar 22;51(3):1–9. doi: 10.1038/s12276-019-0232-4 (PMC6430805; doi:10.1038/s12276-019-0232-4)

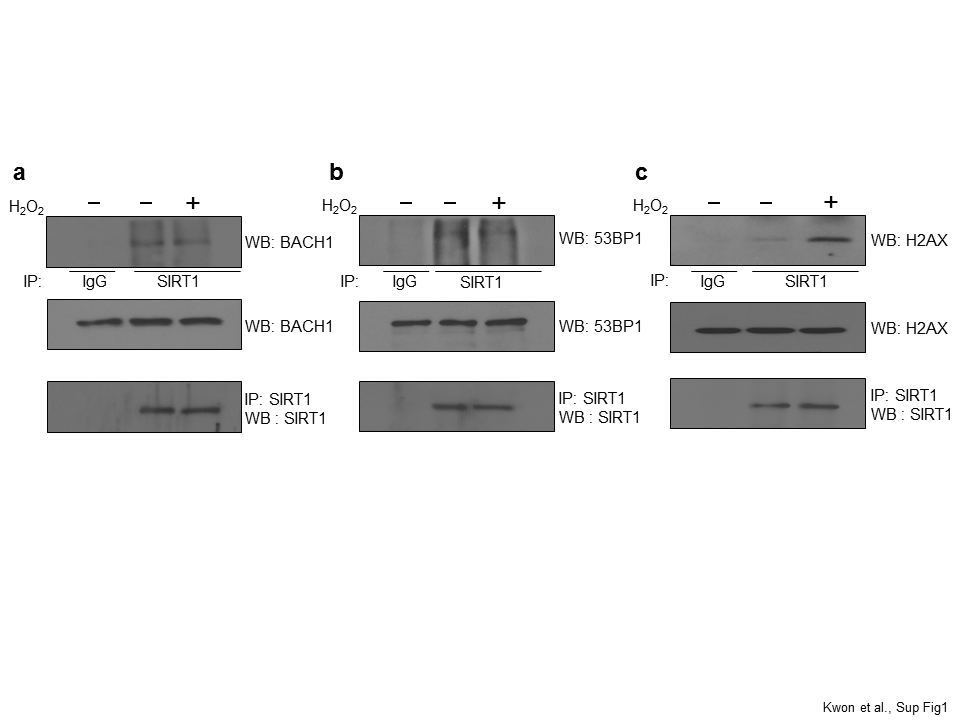

Supplement: Supplementary file 2 — Supplementary Figure 1 [file 12276_2019_232_MOESM2_ESM.tif]

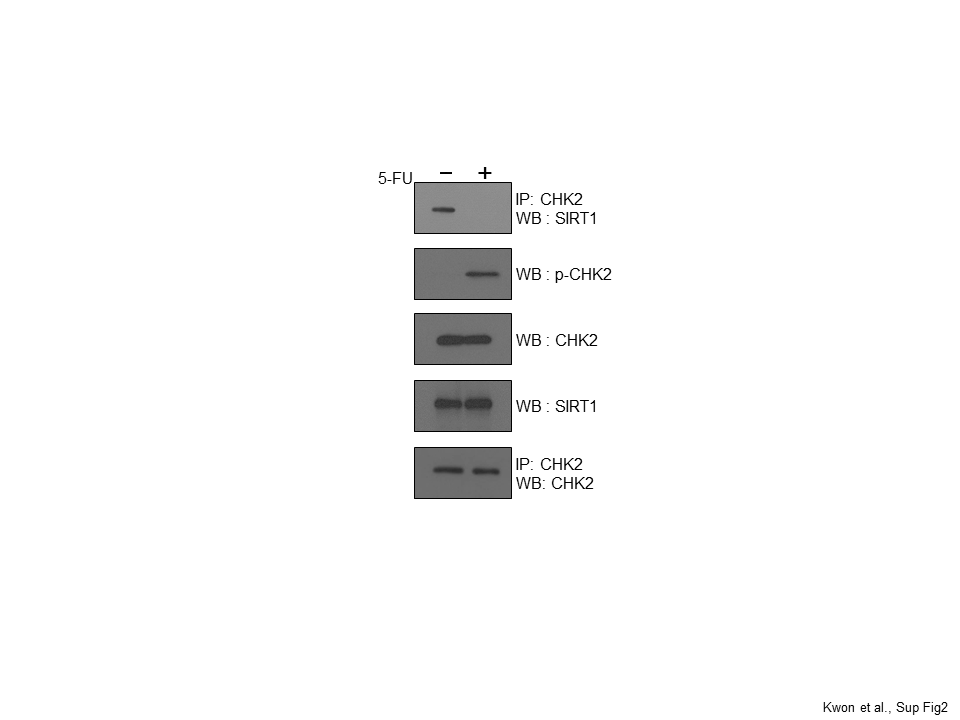

Supplement: Supplementary file 3 — Supplementary Figure 2 [file 12276_2019_232_MOESM3_ESM.tif]
